# Complete Genome Sequence of Amino Acid-Utilizing *Eubacterium acidaminophilum* al-2 (DSM 3953)

**DOI:** 10.1128/genomeA.00573-14

**Published:** 2014-06-12

**Authors:** Anja Poehlein, Jan R. Andreesen, Rolf Daniel

**Affiliations:** a Genomic and Applied Microbiology and Göttingen Genomics Laboratory, Georg-August University, Göttingen, Göttingen, Germany; b Institute of Biology, Microbiology, University of Halle, Halle, Germany

## Abstract

*Eubacterium acidaminophilum* is a strictly anaerobic, Gram-positive, rod-shaped bacterium which belongs to cluster XI of the *Clostridia*. It ferments amino acids by a Stickland reaction. The genome harbors a chromosome (2.25 Mb) and a megaplasmid (0.8 Mb). It contains several gene clusters coding for selenocysteine-containing, glycine-derived, and amino acid-degrading reductases.

## GENOME ANNOUNCEMENT

The strictly anaerobic, Gram-positive, and rod-shaped bacterium *Eubacterium acidaminophilum* belongs to cluster XI of the *Clostridia* (1) and is closely related to *Clostridium sticklandii* and *Clostridium litorale*. This strain is able to conserve energy by degrading amino acids via a Stickland reaction (2). *E. acidaminophilum* is able to use glycine and its derivatives sarcosine (*N*-methylglycine) and betaine (*N*,*N*,*N*-trimethylglycine) as sole energy and carbon sources. This organism was originally isolated from anaerobic black mud from a wastewater ditch (3).

The MasterPure complete DNA purification kit (Epicentre, Madison, WI) was used to isolate chromosomal DNA of *E. acidaminophilum* al-2 (DSM 3953). Sequencing was done by combined approaches of 454 GS-FLX pyrosequencing system (Roche Life Sciences, Mannheim, Germany) and Genome Analyzer II (Illumina, San Diego, CA). Shotgun libraries were prepared according to the protocols of the manufacturers. Sequencing resulted in 2,841,828 reads by Illumina and in 218,691 reads by 454. The *de novo* assembly performed with the Roche Newbler assembly and MIRA 3.4 softwares resulted in 72 contigs. The average coverage is 24.3 (for 454) and 93.7 bp (for Illumina). Gap closure was done by PCR-based approaches, Sanger sequencing of the PCR products, and employing the Gap4 (version 4.11) software of the Staden package (4). The complete genome of *E. acidaminophilum* al-2 (DSM 13864) comprises one circular chromosome (2.25 Mb) and one megaplasmid (0.8 Mb). The overall G+C content is 44.08 mol%. Automatic gene prediction was performed using the software tools YACOP and Glimmer (5). The identification of rRNA and tRNA genes was done with RNAmmer and tRNAscan, respectively (6, 7). The Integrated Microbial Genomes-Expert Review (IMG-ER) system (8, 9) was used for automatic annotation, which was subsequently manually curated using the Swiss-Prot, TrEMBL, and InterPro databases (10). We identified 7 rRNA operons, 68 tRNA genes, 1,911 protein-coding genes with predicted functions, 999 genes coding for hypothetical proteins, and 3 pseudogenes. The genome possesses 8 predicted genes coding for selenocysteine-containing proteins, of which most are involved in the degradation of formate, glycine, sarcosine, and betaine. Genes coding for these multimeric reductases are typically organized in gene clusters. We identified two gene clusters encoding a glycine, two gene clusters encoding a sarcosine reductase, and one gene cluster encoding a betaine reductase. Similar gene clusters were identified in other amino acid-degrading organisms, such as *C. sticklandii*, *Clostridium difficile*, or *Sporomusa ovata* (11, 12). The genome of *E. acidaminophilum* lacks a dihydrolipoamide dehydrogenase (P3 component of glycine decarboxylase) but harbors a unique 11-kDa selenoprotein (PrpU), which forms an operon together with three genes coding for glycine decarboxylase and a formyltetrahydrofolate synthetase. This protein harbors a putative redox active motif (CxxU) and might be involved in electron transfer between glycine decarboxylase and glycine reductase, together with the thioredoxin system (13). The genome contains two clusters coding for formate dehydrogenases, including the catalytic and selenocysteine-containing subunit FdhA. One cluster is associated with genes coding for an iron-only hydrogenase (14, 15).

### Nucleotide sequence accession numbers.

The genome sequence has been deposited in GenBank under accession no. CP007452 (chromosome) and CP007453 (megaplasmid).
